# INTEREST GROUP SESSION—SYSTEMS RESEARCH IN LONG TERM CARE: DEVELOPING AN EVIDENCE BASE FOR NURSING HOME STAFFING IN EUROPE AND BEYOND: MANY PATHS TO ONE GOAL?

**DOI:** 10.1093/geroni/igz038.1395

**Published:** 2019-11-08

**Authors:** Jan Hamers, Ramona Backhaus, Kirsten Corazzini

**Affiliations:** 1 Maastricht University, Maastricht, Netherlands; 2 Duke University, Durham, North Carolina, United States

## Abstract

Despite heterogeneity across countries, nursing homes worldwide have to ensure the delivery of high quality care. At the same time, adequately staffing the homes remains a major concern in most countries. It is a significant challenge to determine the numbers and type of staff as well as staff’s competencies that are necessary to meet the complex needs of nursing home residents. While, especially in the US, research on the relationship between staffing and quality in nursing homes has received considerable attention, the research literature is contradictory. Evidence shows that employing more staff or more registered nurses instead of nurse assistants will not automatically lead to better nursing home quality. This inconsistency of evidence might be explained by a myriad of methodological and theoretical challenges. This symposium aims to provide an international perspective on how researchers from different European countries contribute to the development of an evidence base for nursing home staffing. The first presenter will draw on findings from a realist review conducted in the UK, focusing on the mechanisms (how, why and in what circumstances) under which staff influence care home quality. The second presenter will offer insights into a Swiss study, taking a broader perspective on measuring staffing and quality in nursing homes. The third presenter will present a new instrument to identify staffing ratios in German nursing homes and provide insight into its development process. The fourth presenter will discuss the results of a critical review on the evidence base of a Dutch nursing home staffing guideline.

